# “Leadership in Rehabilitation Teamwork: Challenges for Developing Countries”

**DOI:** 10.3389/fresc.2022.1070416

**Published:** 2022-12-21

**Authors:** Taslim Uddin

**Affiliations:** Department of Physical Medicine and Rehabilitation, Bangabandhu Sheikh Mujib Medical University, Dhaka, Bangladesh

**Keywords:** developing country, leadership in rehabilitation, team leader, rehabilitation team works, rehabilitation health system

## Abstract

Physical rehabilitation medicine is a diverse specialty; its main objective is to provide comprehensive rehabilitation involving multiple health care professionals to optimize function and improve the quality of life for people with disabilities. There is an increase in the number of people with disabilities, and people with disabilities in lower income countries do not receive the required rehabilitation. Rehabilitation intervention includes functional assessment, rehabilitation goal setting, composition of the focused team and coordination of the team works, all of which require a highly skilled team leader. No single professional is likely to have all the necessary skills to achieve optimal results alone. There is a knowledge gap between the theory, existing situation, and practice in rehabilitation team functioning. In this short communication challenges for quality rehabilitation services were highlighted, including the importance of the leadership role of team functioning.

## Introduction

The continued occurrence of injuries and the emergence of noncommunicable chronic diseases increases the number of people with disabilities, particularly in developing countries, and the growing need for rehabilitation remains largely unmet globally (1, 2). People with disabilities suffer the most in low and middle-income countries, where they do not receive the necessary rehabilitation, and the COVID-19 pandemic has further disrupted rehabilitation services (3). So is the necessity to escalate and establish quality rehabilitation services lead by a quality team leader. An attempt was made to analyse the challenges for quality rehabilitation services, with particular emphasis being given on the importance of the leadership role of team functioning.

Medical rehabilitation is a diverse specialty. The conditions commonly dealt with include but are not limited to musculoskeletal disorders including joint problems, neurological disorders including stroke, spinal cord injury, brain injury, cardio-respiratory conditions, metabolic conditions including neuromusculoskeletal complications presenting in acute, postacute or chronic care settings (4).

The rehabilitation interventions include exercises; modifying an individual's home environment; training and education on healthy living; prescribing medicines; providing psychological support; fitting an orthosis, prosthesis, or assistive devices (1). Clearly, these interventions are usually complex in nature, patient-centered team functioning, and require a number of specific tasks and components. It is critical to understand how the rehabilitation team functions and the role of a rehabilitation medicine physician as a leader in low-resource rehabilitation settings.

## Method

This was a short communication based on minireview of the available online literatures made during the period April to Sept 2022. The principal focus was on Developing country, Rehabilitation and Team works.

### Importance and challenge of rehabilitation team working

Effective rehabilitation team working has been recognized and described since World War 1 (5). Teamwork is a working standard in rehabilitation, and it can also be a valid tool to increase the number of professional and sometimes non-professional human resources in the care sector, particularly in developing countries. Different forms of team work, including interdisciplinary (IDR), multidisciplinary (MDR), and transdisciplinary rehabilitation formats, were described. However, there are disputes about the suitability, effectiveness, and setting of each type of team (6, 7, 8, 9). The composition of the rehabilitation teams and the number of team members vary widely depending on the disease condition and the individualized choice of the primary team member. There were dilemmas about the exact nature of the relationship between team members, which was not always specified (6). In some advanced indoor settings of rehabilitation in developed countries, the MDR team must meet every week, which is rare in developing regions (10). A few other countries use a full rehabilitation team that includes a PRM physician, a rehabilitation nurse, orthotics and prosthetics, rehabilitation therapists (physical, occupational, speech language), medical social workers, and other critical-skilled professionals (11, 12).

### Leadership role in rehabilitation team functioning

Effective team management is considered essential for rehabilitation goal setting team meetings and subsequent team coordination. Many of the developing countries' rehabilitation is not a health priority and faces multiple challenges in rehabilitation team functioning (9, 13, 14, 15, 16). Then, what should be the format of the rehabilitation team and who should be leading and coordinating the team functions? Theoretically, personnel who have adequate training, knowledge, and skills required to make a pathologic diagnosis of the condition, evaluate activity and participation restrictions, and be able to select treatment options are required to lead the multidisciplinary rehabilitation team. No single professional is likely to have the full range of skills to achieve optimal results alone. There is a knowledge gap between the theory, the existing situation, and practice in rehabilitation. A good leader must be skilled at clarifying, defining issues, setting goals, and coordinating action steps. He or she should be complying with the mission of the country or the institute at large. The Professional Practice Committee of the Union of European Medical Specialists (UEMS) PRM Section reviewed patterns of teamwork and debated recommendations for good practice during 2008. The consensus statement was that a PRM physician is well-placed to coordinate PRM programs and to develop and evaluate new management strategies (6). There are some success stories of physician-led rehabilitation teams functioning in developing countries' rehabilitation settings (17). In order to stimulate the growth of physical medicine and rehabilitation practices in low-resource nations, organizations like the International Rehabilitation Forum (IRF) established a network of global leaders by offering leadership and specialty training to local practitioners (18). Consequently, one or two physical rehabilitation medicine physicians successfully lead rehabilitation teams in some nations in Asia and the sub-Saharan area. The IRF played a typical neutral catalyst role in transferring rehabilitation leadership thought (9, 16, 18).

Multiple challenges were identified for developing a physician-led rehabilitation team functioning in developing countries presented in Box 1.

BOX 1 **Challenges for leadership role in team functioning [9,13,14, 15,16,19, 20]**

• Lack of relevant course contents of the rehabilitation professional curriculum
• Poor training monitoring system and feedback options
• A scarcity of trained and experienced rehabilitation professionals, a lack of infrastructure development, and inadequate funding in the rehabilitation sector
• Overburdened rehabilitation professionals with poor attitudes toward service providers
• Low rate of education of the persons with disability with political interference of administration
• Potential ethical issues and dilemmas in team functioning
• Minimal or no preparedness for disaster related onslaught casualties

## Conclusion

There is a knowledge gap between the theory, existing situation, and practice in rehabilitation team functioning. Rehabilitation team functions better when it is led by a physiatrist. Multiple challenges exist for developing a physician-led rehabilitation team in developing countries, which may be addressed with local arrangements.
